# Experiences mapping a legacy interface terminology to SNOMED CT

**DOI:** 10.1186/1472-6947-8-S1-S3

**Published:** 2008-10-27

**Authors:** Geraldine Wade, S Trent Rosenbloom

**Affiliations:** 1 Information Management, Hewlett-Packard Company, Atlanta, GA, USA; 2 Department of Biomedical Informatics, Vanderbilt University, Nashville, TN, USA

## Abstract

**Background:**

SNOMED CT is being increasingly adopted as the standard clinical terminology for health care applications. Existing clinical applications that use legacy interface terminology need to migrate to the preferred SNOMED CT standard. In this paper, we describe our experience and methodology for mapping concepts from a legacy system to SNOMED CT.

**Methods:**

Our approach includes the establishment of mapping rules between terminologists and back and forth collaboration of the mapped results through one or more iterations in order to reach consensus on the final maps.

**Results:**

We highlight our results not only in terms of the number of matches, quality of maps, use of post-coordination, and multiple maps but also include our observations about SNOMED CT including inconsistencies, redundancies and omissions related to our legacy mapping.

**Conclusion:**

Our methodology and lessons learned from this mapping exercise may be helpful to other terminologists who may be similarly challenged to migrate their legacy terminology to SNOMED CT. This mapping process and resulting discoveries about SNOMED CT may further contribute to refinement of this dynamic, clinical terminology standard.

## Background

Institutions and Electronic Health Record (EHR) system vendors are being increasingly challenged to use recognized standard terminologies, such as SNOMED CT [1]. Using standard terminologies often requires that system developers migrate away from legacy interface terminologies [2] by mapping them to a reference terminology. Interface terms have largely been proprietary in nature and often used in stand-alone applications that have become outdated or need modification for clinical utility. Using a standard terminology rather than a legacy interface terminology may help make EHR systems be interoperable with other such systems, drive decision support algorithms, enable data aggregation for quality analysis/outcomes measurements, among other tasks [3-5]. Using standardized terminology within applications may support evidence-based initiatives, improve patient safety as well as meet new regulatory requirements [6] as standards are adopted both nationally and internationally. Vanderbilt University Medical Center (VUMC) in Nashville, TN, USA, has developed a clinical interface terminology for use in its EHR system components, including a structured entry tool designed to support clinical documentation. The terminology was designed as an outgrowth of the one created in the 1980s to support the Internist/QMR diagnostic expert and decision support system [7]. The interface terminology includes concepts for general medical evaluation, including those covering history, exam and diagnoses.

## Methods

The terms representing legacy interface concepts were extracted from the Vanderbilt EHR systems in a flat file format (i.e. Excel spreadsheet) for evaluation and mapping. Concepts and their unique identifiers were obtained (e.g. ID02964: *Anaphylactic Shock*) sequenced by a progressive list of concept identifier numbers. No corresponding clinical context from the computer programs using the terminology was initially provided. The concepts related to history (e.g. *Ethanol Dependence History*), history or symptom (e.g. *Myalgia History or Symptom*), physical examination (e.g. *Heart Sound S3 Auscultated*, *Ear Erythema Observed*, *Tactile Fremitus Palpated*), diagnoses (e.g. *Leukemia, Ulcerative Colitis, Sinusitis, Breast Cancer*), time (e.g. *Date of Last Menstrual Period)*, objects (e.g. *Shunt for Hemodialysis Access*, *Implanted Cardiac Device*), procedures (e.g. *Appendectomy, Venous Access Device Placement*), scales (e.g. *Patient Pain Scale, Epworth Sleep Scale Score*) and social (e.g. *Unemployed, Family Makeup*). Several concepts did not appear to fit any particular category or were less well-defined (e.g. *Has a Gun in the House, Wears a Helmet while Riding a Motorcycle*).

Before mapping, there was general agreement between the two terminologists on mapping rules including how the quality of the mapping relationships would be defined (see below) and how post coordinated concepts would be represented.. For example, several source concepts were entities that were "auscultated" (e.g. *Heart Murmur Auscultated*, *Abdominal Bruit Auscultated*). These were all to be mapped similarly using agreed upon post-coordinated concept groupings in SNOMED CT (e.g. Finding by auscultation [finding] Associated with [attribute]).

The concept mapping process involved 4 steps. The first step was to group the legacy (source) concepts into relevant clinical categories (Table 1). Concepts that included terms such as *Auscultated *or *Palpated *were grouped similarly and were assessed as being part of a physical examination.

**Table 1:** Grouping of legacy interface terminology concepts with corresponding examples

**CATEGORIES** **(# of concepts)**	**LEGACY CONCEPTS**
**Historical (500)**	
**Allergy (12)**	ALLERGY TO LATEX
**Family History (40)**	FAMILY HISTORY OF NEUROPATHY
**History (80)**	DYSLEXIA HISTORY
**History or Symptom (353)**	DIAPHORESIS HISTORY OR SYMPTOM
**Ob-Gyn History (8)**	NUMBER OF CHILDREN
**Risk Factors (3)**	CARDIAC RISK FACTORS
**Physical Exam (972)**	
**Auscultation (68)**	VENOUS HUM AUSCULTATED
**Elicited (200)**	PULSUS PARADOXUS ELICITED
**Measurement (56)**	BODY MASS INDEX QUANTITATIVE MEASURED
**Observation (487)**	AGITATION OBSERVED
**Palpation (148)**	HEART THRILL PALPATED
**Percussion (13)**	LIVER SPAN QUANTITATIVE PERCUSSED
**Other (530)**	
**Activities and Functions (49)**	USE OF AMBULATION ASSISTIVE DEVICES
**Chief Complaint (12)**	CHIEF COMPLAINT EXPOSURE TO CHEMICAL
**Clinical finding (299)**	MENINGITIS
**Date (56)**	DATE OF FIRST POLIO VACCINATION
**Devices (5)**	INDWELLING URINARY CATHETER
**Misc. (7)**	PATIENT TRANSFERRED FROM
**Personal and Social (27)**	TYPE OF LIVING ACCOMODATION
**Procedure (44)**	REPAIR OF TETRALOGY OF FALLOT
**Referral (5)**	REFERRAL FOR ABNORMAL ECHOCARDIOGRAM
**Scales and scores (26)**	EPWORTH SLEEP SCALE SCORE

Those concepts that included *History, History or Symptom, Family History, Risk Factors*, etc. were also grouped similarly and were assessed as being historical. Some concepts were grouped based on the terminologists' judgment that included underlying clinical knowledge/domain expertise. For example, concepts such as *Supports Self on Forearms While Prone *and *Plays "Pat-A-Cake" Responsively *were known to be observations of one's development status and *Inguinal Herniorrophy *and *Mastectomy *were known surgical procedures. A concept such as *Taking Anticoagulant Medication *could have been placed in more than one grouping (e.g. History or Activities and Functions) but a single group (i.e. Activities and Functions) was subjectively selected for mapping purposes. Some of the concepts were categorized as miscellaneous when they did not appear to be part of logical group (e.g. *Patient Transferred From*, *Follow Up Evaluation For*, etc). By grouping the concepts in this way, most could be correlated with the upper level SNOMED CT categories/axes. Additionally, groups of similar concepts could be mapped in a consistent way using similar rules. This was most important for representing SNOMED CT concepts requiring post-coordination.

The second step involved searching the SNOMED CT knowledgebase (January 2005) [8] for concepts within each of the groupings. Both proprietary search tools [9] and the Clue Browser [10] were used. Concepts were searched for and selected by using their word matching and/or synonym matching with consideration of where they fit within in a given hierarchy. If the source concept was a procedure, a corresponding target concept in the SNOMED CT procedure axis was selected.

The third step was to record the selected target concepts in a spreadsheet adjacent to the source (legacy) concept. Only active non-limited SNOMED CT concepts were selected as targets. The target concept used in the result set included the fully specified name designated by SNOMED CT. As each map was recorded, a separate entry was also recorded as to the quality of the relationship between and source legacy interface terms and target SNOMED CT concepts. A source concept that mapped to a semantically equal single SNOMED CT concept was qualified as equal.

An equal qualifier was also given to maps that used combined target concepts using the post coordination guidelines developed by the SNOMED CT Concept Model Working Group [11], the SNOMED CT Users guide [12] and the Technical Implementation guides [13].

They were noted under a separate category (see Results, Table 2). The same was done for relationships that were qualified as related but not equal to a single target concept or targets. A source concept that was not mappable to target concepts in SNOMED CT was recorded as "No Match". Some final maps included IS A relationships since the source concept only appeared to relate to higher-level concepts in SNOMED CT.

**Table 2:** Results of legacy concept mapping to SNOMED CT with examples

Number of concepts (2002 = total)	Mapped relationship(s)	Source concept(legacy)	Target concept (SNOMED CT)
1510 (75%)	302 mapped to equal single target concept	OSTEOPOROSIS	Osteoporosis (disorder)
	1208 mapped to equal post-coordinated targets	POST-ICTAL PSYCHOSIS	Psychotic disorder (disorder) Associated with (attribute) Post-ictal state (finding)
396 (20%)	34 mapped to related single target concept	BEHAVIORAL DISTURBANCE	Problem behavior (finding)
	362 mapped to related post-coordinated targets	PELVIS MUSCULAR TONE FLACCID PALPATED	Finding by palpation (finding) Associated with (attribute) Poor pelvic muscle tone (finding)
70 (4%)	Mapped to parent target concept only (IS A)	SMILES TO IMAGE OF PARENTS FACE	Child developmental finding (finding)
26 (1%)	No map	HEART SOUND CLICK AUSCULTATED	

The fourth step was to share the resulting groups of maps with the second terminologist for validation and commentary. Each concept map was agreed to or was commented upon for further review/discussion. The maps were then returned to the first terminologist. Comments included requests for remapping, additional clarification as to why a given target was chosen and clinical explanations as to why the SNOMED synonym was incorrect or inconsistent. On occasion, additional context was provided to the first terminologist based on knowledge of the actual clinical context. For example, the concept *Orthopedic Surgery *could be interpreted as referring to the Orthopedic Surgery Department or to an Orthopedic surgical procedure.

The process of back and forth collaboration between the two terminologists (GW, STR) continued for two or three iterations until all maps were completed.

## Results

**Figure 1:**
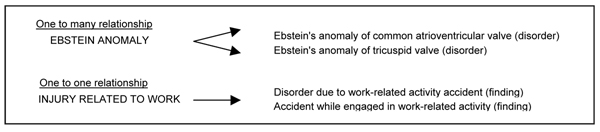
Mapping examples showing different relationships to target maps.

2002 legacy interface terms from VUMC were evaluated. Among the resulting final maps to SNOMED CT (Table 2), there were 1510 concepts that were rated by two terminologists (GW, STR) as having semantically equivalent matches. In this group, 302 legacy concepts mapped each to single SNOMED CT concepts and 1208 legacy concepts mapped to a combination of post-coordinated concepts. Maps that were related but not semantically equal included 34 single concept maps and 362 post-coordinated maps. Seventy concepts were designated as having an IS A relationship as they appeared to represent an appropriate child concept relative to a SNOMED CT concept. Twenty-six concepts were not matched (e.g *Heart Sound Click Auscultated, Presyncope, Low Pitched Bowel Sounds Auscultated*,). Among the post-coordinated maps, 580 were more complex in that several attribute-value pairs were used (e.g., *Precordial Cardiac Impulse Intensity Palpated *mapped to Finding by palpation (finding) + Associated with (attribute) + Finding of pulse volume (finding) + Interprets (attribute) + Precordial pulsation, function (observable entity)). Additional results showed that 9 of the legacy concepts mapped to more than one SNOMED CT concept (i.e. 1 to many relationship). In some instances, there were maps to two equivalent SNOMED CT concepts (i.e. 1 to 1 relationship) Fig. 1

In addition, some of the SNOMED CT target concepts seemed to be formatted inconsistently (e.g. Left popliteal artery structure (body structure) and Structure of right popliteal artery (body structure)). All of the final outputs were recorded in a flat file/Excel spreadsheet and given to the IT group for future consideration/integration into the current clinical application.

## Discussion

SNOMED CT is a dynamic, scientifically validated clinical health care terminology and infrastructure [14] that is being increasingly adopted as the preferred terminology for the representation of clinical information. As healthcare providers, payers and government officials focus on developing interoperable electronic health networks, data standards including SNOMED CT are being increasingly incorporated into new and existing healthcare applications to meet data sharing needs. Transforming legacy and proprietary terminologies into standards will be required for clinical utility. Such legacy interface terminologies, like the one we have described, may consist of an aggregate of single concepts or concept phrases and not part of a structured, controlled terminology. Thus alignment methods that have been described previously [15-17] using algorithms to compare structured knowledge sources could not be used. Formal definition description logics (DLs) have also been shown to aid in mapping between terminologies by providing concept and role definitions with explicit semantics [18]. These, too, were absent from the legacy interface terminology in this evaluation. To offset some of these limitations, we felt that it was important to group concepts into clinically relevant categories ahead of the actual mapping in order to provide some consistency for mapping of concepts within a given group. Lexical associations (i.e. *auscultated, palpated, history or symptom*, etc) included in many of the concept strings helped guide some of the obvious groupings. The terminologists could then discuss and agree prospectively on mapping rules that would apply generally as well as to the differing groups of concepts

After establishing agreed-upon mapping rules, there were a series of process steps involving searching, recording and qualifying relationships among the mapped concepts. There was ongoing collaboration – validation, discussion and commentary for each group of maps. This was critical to achieving eventual consensus on the final maps.

In our experience, it is critical to have terminologists with considerable clinical background or domain expertise who could apply their knowledge to the grouping and mapping of concepts whose meaning may not be obvious by the description alone. In this evaluation of legacy interface concepts, no corresponding clinical context was given ahead of the first mapping iteration and this led to some initial errors. Perhaps by providing some clinical context with a list of legacy concepts there would be better semantic maps with SNOMED CT. By grouping legacy concepts into similar categories prospectively and by using mapping rules in a consistent manner to each group, future changes made to SNOMED CT may be more readily applied to your mapped legacy terminology (e.g. If new attribute-value pairs are added or previous guidelines revised, new pairs of concepts can be consistently applied.)

We observed that this process exposed not only differences between the two terminologists in their semantic interpretation of concepts but also highlighted areas in SNOMED CT that were redundant, inadequate or deficient. For example, we did not think that "depression (finding)" and "sadness" were semantically equal as defined by SNOMED CT. We found that "rectocele" was used as a synonym for the preferred display concept of "female proctocele without uterine prolapse (disorder)", even though there are rare instances when it occurs in a male. This example also highlighted the discovery that some of the preferred display concepts led to a change in a map upon review. Even though there may have been an exact match to a synonym in SNOMED CT, the preferred display concept, on occasion, suggested an alternate meaning that led to a re-examination of the map. This mapping exercise also led to the identification of concepts that needed to be added to SNOMED CT. Despite these deficiencies and omissions, there was overall good clinical concept representation of this legacy interface terminology set in SNOMED CT. Also, it is useful to note that SNOMED CT is dynamic – a work in progress – with biannual updates and new releases. As a standards organization, it is open to participation and invites submissions for additions and modifications. SNOMED CT editors rely on inputs from users. This makes it most suitable for the complexities of clinical medicine. Efforts to extend terminologies such as SNOMED CT into ontologies offer additional sources of discriminating reviews [19,20].

Future consideration of these maps may involve integration of the SNOMED CT terminology into the application interface or in a cross-referencing table. It may be that exact concept matches will have the most immediate potential for integration. Further investigation, i.e. comparing how many exact concept matches correspond with the frequency of clinically used terms in the actual legacy application, may give further insight as to how it may be best to proceed with integration. For instance, a more frequently used clinical concept such as "myocardial infarction" is well represented in SNOMED CT [21] and could be immediately deployed for use within an application. A less frequently used concept, such as "Epworth sleep scale score" is not currently represented in SNOMED CT but may not be critical data for capture as a "standard" as it would be much less likely to be used in decision-support algorithms or patient safety measures.

## Conclusion

Using these 2002 concepts as a typical example of what other terminologists may face when challenged with transitioning their proprietary concepts to standardized terminology, this methodology can be applied using a systematic approach – starting with legacy concept grouping and establishment of rules for mapping concepts that are grouped similarly as well as establishing consensus (between terminologists) for how rules will be applied and for how Attribute-Value pairs will be applied to particular groups of concepts. Such mapping and analysis contributes to the improvements in SNOMED CT as clinical concepts are continuously added and modified (through submissions and inquiries).

## Competing interests

The authors declare that they have no competing interests.

## Authors' contributions

Both authors (GW and STR) designed the study, performed and evaluated the concept mappings, drafted and approved the final manuscript.
